# Changes in mental disorder prevalence among conflict-affected populations: a prospective study in Sri Lanka (COMRAID-R)

**DOI:** 10.1186/s12888-015-0424-y

**Published:** 2015-03-10

**Authors:** Chesmal Siriwardhana, Anushka Adikari, Gayani Pannala, Bayard Roberts, Sisira Siribaddana, Melanie Abas, Athula Sumathipala, Robert Stewart

**Affiliations:** 1 Faculty of Medical Science, Anglia Ruskin University, Chelmsford, UK; 2 King’s College London (Institute of Psychiatry), London, UK; 3 Institute for Research and Development, Colombo, Sri Lanka; 4 London School of Hygiene and Tropical Medicine, London, UK; 5 Department of Medicine, Faculty of Medicine and Allied Sciences, Rajarata University of Sri Lanka, Anuradhapura, Sri Lanka; 6 Research Institute for Primary Care and Health Sciences, Keele University, Keele, UK

## Abstract

**Background:**

Longitudinal data are lacking on mental health trajectories following conflict resolution and return migration. COMRAID-R is a follow-up study of Muslims displaced by conflict from Northern Sri Lanka 20 years ago who are now beginning to return.

**Methods:**

Of 450 participants in displacement interviewed in 2011, 338 (75.1%) were re-interviewed a year later, and a supplementary random sample (n = 228) was drawn from return migrants with a comparable displacement history. Common mental disorder (CMD; Patient Health Questionnaire) and post-traumatic stress disorder (CIDI-subscale) were measured.

**Results:**

A CMD prevalence of 18.8% (95%CI 15.2–22.5) at baseline had reduced to 8.6% (5.6–11.7) at follow-up in those remaining in displacement, and was 10.3% (6.5–14.1) in return migrants. PTSD prevalences were 2.4%, 0.3% and 1.6% respectively.

**Conclusions:**

We observed a substantial decrease in CMD prevalence in this population over a short period, which may reflect the prospect of return migration and associated optimism following conflict resolution.

## Background

Conflict-driven forced migration results in the displacement of millions of people around the world both within and across national borders, the majority of whom are non-combatant civilians. There are an estimated 28 million internally displaced persons (IDPs) globally and these are particularly vulnerable, lacking the international legal protection afforded to refugees [1]. However, trauma is shared by refugees and IDP alike. The negative mental health impact of conflict-driven forced displacement has been widely researched and documented [2,3].

Porter and Haslam [2] exploring pre-displacement and post-displacement factors associated with the mental health of refugees and IDPs in a meta-analytic review, found that factors such as the type of accommodation during displacement, post-displacement economic opportunities, cultural access, conflict status, age, gender differences and pre-displacement urban–rural residence were associated with mental health outcomes to a moderate extent. They concluded that development of psychopathology among refugees and IDPs was not wholly accounted for by exposure to a traumatic experience such as conflict and related unavoidable post-traumatic sequelae, but instead involved a wider combination of contextual, cultural, social and economic factors associated with predisplacement and postdisplacement periods [2]. Another systematic review of factors influencing psychological health of LMIC populations affected by conflict concluded that female gender, low education, low economic status, unemployment, camp residence, poor living conditions, security-related issues and number of traumatic experiences are associated with poor psychological health in these populations [3].

These systematic reviews and other evidence show that the research on mental health impact of forced displacement has been limited by a lack of longitudinal data, a lack of evidence on prolonged displacement and a focus on a narrow spectrum of disorders such as post-traumatic stress disorder (PTSD) and depression rather than wider common mental disorders (CMDs) [2-4] including somatoform disorder, anxiety disorder, mood disorders (major and other depression) [5].

The mental health impact of return migration has received little research, particularly in IDP populations experiencing prolonged displacement [3,6]. Forced displacement episodes can be of short or long duration, depending largely on the original trigger event [3]. Some episodes end within weeks or months, enabling a rapid return of the affected populations to areas of origin, while others involve displacement for decades [7]. While initial migration is generally unavoidable in conflict-driven situations [7], return migration following conflict resolution often presents a difficult choice if displacement has been prolonged: between a desire to return to an area of origin, and the reality of partial or total settlement in the displacement area [6,7]. Although return migration is sometimes imposed by political pressure [8], prolonged forced displacement is known to discourage return migration, especially if acculturation has taken place and/or if new generations have been born during the post-flight period [7,8]. Return migration may be influenced by the nature of displacement [7-9], specifically the nature and level of trauma experienced during the original displacement [10] and by other factors including assurance of security, availability of livelihood and service availability at the destination [3]. Levels of acculturation, expectations on ‘return’, generational divisions within families or communities, and potential re-traumatisation can act as ‘push and pull’ factors influencing return migration in post-conflict situations [3,7].

The available evidence suggests that conflict-related psychopathology tends to persist for several years, compounded by limited healthcare and reconstruction efforts in former conflict areas [11]. Although evidence from refugee populations suggests that prolonged displacement in favourable conditions may predict better mental health outcomes [3], IDPs may be particularly vulnerable to adverse mental health outcomes, in part due to low-resource, camp-like settings and other daily stressors [6]. A recent study in severely conflict-affected districts of Sri Lanka showed that long-term displacement in IDP camps is negatively associated with well-being and mental health [12]. Despite the critical public health importance of conflict-affected forced migration, the lack of a comprehensive evidence base is a major obstacle in understanding risk and protective factors related to mental health issues around the return migration process, especially important in a rapidly changing world where the ability to migrate may have substantial social and economic benefits [11,13].

The ‘**CO**mmon **M**ental Disorders and **R**esilience **A**mong **I**nternally **D**isplaced in Sri Lanka – follow up study on **R**eturn migration (COMRAID-R)’, is a follow-up study investigating mental health outcomes in a specific population of IDPs affected by prolonged (over 20 years) forced displacement as a result of conflict in Sri Lanka. This population of IDPs numbered about 75,000 at the time of displacement, belonged to an ethnic Muslim minority (a distinct ethnic group of Islamic faith in Sri Lanka mainly speaking Tamil language) and were forced to leave northern Sri Lanka due to conflict in 1990 [14,15]. Following conflict cessation in 2009, they are currently considering or engaging in return migration to areas of origin [14]. Having initially found a high prevalence of CMD in this population [16], we sought to investigate changes in CMD and PTSD prevalence during the post-conflict period and to compare return migrants with those continuing to live in displacement.

## Methods

### Study design

The baseline COMRAID cross-sectional survey was carried out in 2011, sampling a population of ethnic Muslim IDPs who had been forced to relocate in 1990 from Mannar district in Northern Province to Puttalam district in North-Western Province of Sri Lanka [16]. Overall CMD prevalence was 18.8% in the baseline sample, somatoform disorder 14.0%, major depression 5.1%, and PTSD 2.8%. Significant associations were found between CMD and unemployment, widowed/divorced status and food insecurity [16]. The follow-up (COMRAID-R) was carried out in 2012, one year later, and involved tracing and re-interviewing previous participants as well as recruiting a supplementary sample of return migrants from this particular population who were now resident in Mannar district, the original location from where they had been displaced.

The background to this particular community, including their displacement history, has been previously described in detail [14-16]. In summary, participant communities had experienced prolonged conflict-initiated displacement for over 20 years and included generations born during the post-flight period. At the time of the baseline study, 2 years had elapsed since the end of conflict in Northern Sri Lanka, but return migration had not started to any significant degree. This was because the region was undergoing land mine clearance and infrastructure re-development work, as 30 years of conflict had completely destroyed health, educational, transport and other systems. When the baseline study was being conducted, the IDP community did not have any information about when or if they would be able to return to the area of origin in Mannar after their 20 year exile [14]. By the time of the follow-up study described here (3 years after conflict cessation), return migration had officially started, and IDPs were being encouraged to return to Mannar by the national government. However, uncertainty still prevailed, as available guidance and information on return-relevant factors (e.g. schedule, support from government, available resources, land reclamation and re-allocation, security and civil administration) was conflicting and scarce. In addition, there were new generations born in displacement with no knowledge of the ‘area of origin’. For these reasons, many of the baseline study population had opted to remain in displacement, or had not yet been drafted in to the process of return migration by the government – hence the need for a supplementary sample of return migrants at follow-up.

Ethical approval for the study was obtained from the Psychiatry, Nursing and Midwifery Research Ethics Subcommittee of King’s College London and the Ethics Review Committee, Faculty of Medicine and Allied Sciences, Rajarata University of Sri Lanka. Informed written consent was obtained from each participant. Ethical and cultural challenges in conducting both COMRAID 1 and 2 have been previously described [17].

### Sampling

Participants were recruited from two sources: 1) participants from the baseline COMRAID study (n = 450) who were traced and approached for re-interview; 2) a supplementary random sample drawn from residents in Mannar district who had previously resided in Puttalam resettlement units and had subsequently returned. For the supplementary sample, the same inclusion criteria were applied as in the baseline sample (Sri Lankan nationality, aged 18–65, originally resident in the Northern Province of Sri Lanka, displaced in 1990 and subsequently residing in welfare camps and other settlements in Kalpitiya division of Puttalam district, or born to at least one displaced parent with these characteristics). In addition, at least six months residence in northern Sri Lanka after return migration was required. As indicated in the inclusion criteria, participants were either displaced themselves or were born into displaced families.

**Figure 1:**
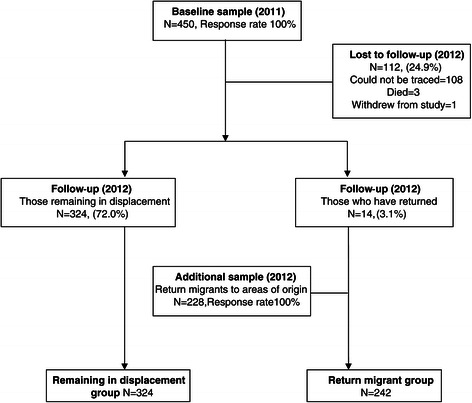
**Sampling methodology flow diagram.**

A multi-stage sampling strategy adopted and used successfully during the baseline study was utilised to recruit the additional sample of returnees (See Figure 1) [16]. This comprised random selection of Grama Niladhari divisions (GNDs; the smallest civil administrative division in Sri Lanka) using government lists from Mannar district, based on probability proportionate to each village population. All selected GNDs were areas of origin for the IDP populations of interest. Ten households of return-migrant families were then randomly selected using government-provided information. Finally, a participant meeting inclusion criteria was randomly selected from each household using the KISH method [18]. The additional sample of returnees recruited at follow-up had thus been living in displacement in Puttalam district for the same length of time, experiencing similar camp/settlement conditions. Earlier return had been possible due to certain areas being cleared of mines earlier, due to their camps being selected by the government for resettlement earlier than others (which was linked to the progress of mine clearing) and due to the step-wise nature of government allocation of land and other resources [14]. In the additional sample of returnees recruited from Mannar for COMRAID-R, the mean number of years since return was 2.2 (SE 0.6; range 0–4).

### Measurements

The follow-up study comprised structured interviews, with trained research assistants administering identical instruments to those completed at baseline on socio-demographic information and mental health. Common mental disorders were ascertained using the Primary Care Evaluation of Mental Disorders Patient Health Questionnaire (PRIME-MD PHQ) which measures current prevalence, and the K-section of the Composite International Diagnostic Interview (CIDI-K) was used to ascertain PTSD prevalence [16,19,20]. Tamil language versions of both instruments had been used previously in the same population (PHQ cronbach alpha; 0.861, CIDI K cronbach alpha; 0.755) [16]. In addition, both instruments have been utilised in a national mental health survey in Sri Lanka and adapted by using a combination of quantitative and qualitative methods that emphasised its cross-cultural equivalence [21,22].

In addition to information on age (current age in relation to displacement event in 1990 - see Table 1), gender, marital status, ethnicity, education, current debt, food insecurity (defined as more than 60 days in the last calendar year lacking sufficient food to meet household requirements) and employment, new questions were administered ascertaining household assets, land ownership and source of resettlement land. The household asset index was used as a proxy measure of household income and economic status, based on an index previously developed and used in Sri Lanka [23] and encompassing 8 assets: i) source of drinking water, ii) type of toilet, iii) floor, iv) wall and v) roofing material, vi) cooking fuel, vii) ownership of a radio, TV or telephone and viii) vehicle ownership. Each of the 8 assets included three categories that reflected low, middle and high income (for example, type of toilet was categorised into no latrine, pit latrine and water-sealed cistern with scores of 1–3 in ascending order). The scores of each subcategory was then summed up to obtain a total asset score, which was divided by tertiles to classify low, middle and high income for both follow-up and return groups. Family ownership of private land prior to displacement was ascertained (for generations born after displacement, ownership of land by displaced parents/grandparents). Return migrants were also categorised into those resettling to family-owned or government-allocated land.

**Table 1:** **Socio-demographic information of analysed groups**

Characteristic	1. Baseline sample (2011)	2. Previous participants still displaced in 2012	3. Return migrants recruited in 2012	Difference between groups 2 and 3 (chi ^ 2 ^ , df, p-value)
	N = 450	N = 324	N = 242	
**Age**				**0.30 (1), p = 0.028**
***18–21 (born in displacement)***	39 (8.6)	18 (5.7)	22 (9.1)	
***22–37 (child at displacement)***	189 (42.0)	158 (48.8)	93 (38.4)	
***38–65 (adult at displacement)***	222 (49.3)	148 (45.7)	127 (52.5)	
Gender				0.002 (1), p = 0.891
***Male***	166 (36.8)	112 (34.6)	85 (35.1)	
***Female***	284 (63.2)	212 (65.4)	167 (64.9)	
Ethnicity				-
***Muslim***	426 (94.7)	306 (94.4)	242 (100.0)	
***Other (Sinhala/Tamil)***	24 (5.3)	18 (5.6)	0 (0.0)	
Religion				-
***Islam***	424 (94.2)	305 (94.1)	242 (100.0)	
***Other (Buddhist/Hindu/Christian)***	26 (5.8)	19 (5.9)	0 (0.0)	
Marital status				0.07 (1), p = 0.108
***Married***	345 (76.6)	269 (83.0)	186 (76.8)	
***Widowed/Divorced***	37 (8.2)	30 (9.3)	25 (10.3)	
***Never married***	67 (14.8)	25 (7.7)	31 (12.8)	
Education				0.05 (2), p = 0.664
***Primary (Up to grade 5)***	115 (25.6)	72 (22.2)	58 (24.0)	
***Secondary (Up to OL)***	272 (60.7)	206 (63.6)	145 (59.9)	
***Post-secondary (AL & above)***	61 (13.6)	46 (14.2)	39 (16.1)	
Employment				0.002 (1), p = 0.771
***Employed***	182 (40.4)	124 (38.3)	90 (37.2)	
***Unemployed***	268 (59.6)	199 (61.4)	152 (62.8)	
Financial debt				1.45 (1), p = 0.007
***No debts***	256 (57.1)	240 (74.1)	208 (85.9)	
***Indebted***	193 (42.9)	83 (25.6)	34 (14.1)	
Food security (last year)				10.35 (1), p = <0.001
***Sufficient food***	319 (70.1)	256 (79.0)	229 (94.6)	
***Lack of sufficient food***	130 (28.9)	67 (20.7)	13 (5.4)	
Household assets				62.36 (2), p = <0.001
***Low***	-	5 (1.5)	51 (21.1)	
***Middle***	-	171 (52.7)	174 (71.9)	
***High***	-	148 (45.7)	12 (7.0)	
Land ownership				0.76 (1), p = <0.001
***Own land before displacement***	-	82 (25.3)	138 (57.0)	
***Did not own land***	-	239 (73.7)	103 (42.6)	
Resettlement land				-
***Resettled in own land***	-	-	120 (49.5)	
***Resettled in allocated land***	-	-	121 (50.0)	
Social network				0.008 (1), p = 0.873
***Social isolation***	74 (16.4)	44 (13.5)	34 (14.1)	
***Adequate network***	376 (83.6)	280 (86.5)	208 (85.9)	

Note: Group 1 is made of baseline sample participants recruited in Puttalam district in 2011. Group 2 is made of 324 baseline participants traced and re-recruited from Puttalam district in 2012. Group 3 is made of 14 baseline participants who had returned to areas of origin in Mannar district and 228 new recruits from Mannar district.

Social networks were measured by using the abbreviated Lubben Social Network Scale (LSNS-6), which identifies persons at-risk for social isolation [24,25]. The scale is based on three questions (How many relatives do you see or hear from at least once a month? How many relatives do you feel close to such that you could call on them for help? How many relatives do you feel at ease with that you can talk about private matters?) that evaluate social ties among kin (family/relatives) and an identical three questions evaluating social ties among non-kin (friends). Each question is scored on a Likert scale and the total score ranges from 0–30 with higher scores representing increased social contacts, and a cut-point of 12 and below defining those at-risk for social isolation [25]. Both the Tamil language version used in the study sample (Cronbach alpha of 0.73) and the original English version of LSNS-6 (Cronbach alpha of 0.83) had adequate internal consistency [25]. Using the pre-defined cut-off, a binary variable of adequate social networks and risk of social isolation was used.

### Statistical analysis

Double data entry was conducted using the Statistical Package for Social Science (SPSS) software version 17.0. All data analyses were conducted using STATA version 11, and the dataset was adjusted for cluster sampling design and weighted appropriately. For the purpose of data analysis, baseline participants who had completed return migration were grouped together with the supplementary sample of return migrants, as they were too small in size to analyse separately. The remaining group consisted of baseline participants continuing to live in Puttalam district, the region of displacement. Socio-demographic factors and mental disorder prevalence were described for both groups, with prevalence differences calculated between baseline, follow-up and return migrant groups. Relative risk ratio (RRR) was calculated for any CMD, between groups remaining in displacement and returnees, adjusted for socio-demographic factors (gender, age at displacement, marital status, education, food security, financial debt, employment, and social networks). Unadjusted logistic regression analyses were conducted to investigate factors associated with CMD and subgroup disorders in the samples. Significant colinearity was observed between unemployment and gender (Spearman r = 0.718). Therefore, adjusted multivariable logistic regression analyses were carried out for all groups based on two separate models. Model one included CMD, age at displacement, gender, marital status, education, food security, financial debt and social network variables. The second model comprised of CMD, age at displacement, gender, marital status, education, food security, financial debt, social networks and employment variables.

## Results

Of the baseline cohort of 450 who had been recruited with a 100% participation rate, 338 (75.1%) were successfully traced and interviewed with a mean (SE) age of 38.5 (0.65) years. Of these, 14 (3.1%) had returned to Mannar district and the remainder continued to be resident in Puttalam. For the supplementary sample, 228 return migrants in Mannar district were identified and approached, all of whom agreed to participate, resulting in a combined sample of 242 return migrants with a mean (SE) age of 40.4 (0.85) years. Of the 112 baseline participants lost-to-follow-up, 108 could not be traced, 3 had died and 1 withdrew from the study; the mean (SE) age in this group was 36.0 (1.21) and 55% were female. Attrition was positively associated with never-married status (OR 2.1 95%CI 1.2–3.7) and negatively associated with having any CMD at baseline (OR 0.5 95%CI 0.2–0.9) and employment (OR 0.5, 95%CI 0.3–0.8). It was not significantly associated with any other socio-demographic measure at baseline (all p-values >0.05).

Demographic and socio-economic characteristics of the two COMRAID-R samples are summarized in Table 1 with baseline COMRAID sample characteristics included for reference. Gender, education, marital status and employment were similar between those remaining in displacement and return migrants but the age distribution differed, with higher proportions of both youngest and oldest age groups in the return migrant group. Reported debt and food insecurity had fallen substantially in prevalence between baseline and follow-up in the community remaining in displacement, and were lower still in the return migrant group. Return migrants were more likely to have owned land prior to displacement than those who remained in displacement.

Prevalences of mental disorders in the comparison groups are summarized in Table 2. Overall CMD prevalence was substantially lower in the follow-up sample remaining in displacement than it had been at baseline, falling from 18.8% to 8.6%; the prevalence was slightly higher (10.3%) in return migrants, although confidence intervals overlapped. Similar patterns were evident for CMD constituent disorders and for PTSD. Statistically significant prevalence differences were observed for any CMD, somatoform disorder, major depression, other depression and PTSD between the baseline group and the group remaining in displacement. Statistically significant prevalence differences were also observed for any CMD and major depression between the baseline and return migrant groups (Table 2). For illustrative purposes, Table 2 also presents the prevalences of constituent disorders within those who were classified with any CMD. In those with CMD, anxiety disorder showed the most pronounced reduction from baseline to follow-up, and somatoform disorder, other depression and PTSD were more predominant in return migrants than those remaining in displacement.

**Table 2:** **Prevalence of mental disorder in IDPs at baseline, in those remaining in displacement and return migrants at 12 month follow-up**

Mental disorder	Prevalence % (95% CI)			Differences in prevalence (95% CI)*		
	1. Baseline sample (2011)	2. Previous participants still displaced in 2012	3. Return migrants recruited in 2012	Groups 1 and 2	Groups 1 and 3	Groups 2 and 3
***Total sample***	*(N = 450)*	*(N = 324)*	*(N = 242)*			
**Any CMD**	18.8 (15.2–22.5)	8.6 (5.6–11.7)	10.3 (6.5–14.1)	**-10.2 (-14.9,-3.4)**	**-8.5 (-13.7,-3.2)**	-1.7 (-6.5, 3.1)
**Somatoform**	14.0 (10.7–17.9)	5.9 (3.3–8.4)	9.5 (5.8–13.2)	**-8.1 (-12.2,-4.0)**	-4.5 (-9.3, 0.3)	-3.6 (-8.0, 0.8)
**Major depression**	5.1 (3.2–7.7)	2.2 (0.6–3.8)	0.8 (0.03–1.9)	**-2.9 (-5.4,-0.3)**	**-4.3 (-6.6,-1.9)**	-1.4 (-3.3, 0.5)
**Other depression**	7.3 (5.3–10.3)	2.5 (0.5–3.2)	4.1 (1.6–6.4)	**-4.8 (-7.7,-1.8)**	-3.2 (-6.6, 0.2)	-1.6 (-4.6, 1.4)
**Anxiety**	1.3 (0.4–2.9)	0.3 (0.01–0.9)	0.4 (0.04–1.2)	-1.0 (-2.2, 0.2)	-0.9 (-2.2, 0.4)	-0.1 (-1.1, 0.8)
**PTSD**	2.4 (1.2–4.3)	0.3 (0.01–0.9)	1.6 (0.4–3.2)	**-2.1 (-3.6,-0.5)**	-0.8 (-2.9, 1.3)	-1.3 (-2.9, 0.3)
CMD present**	*(N = 85)*	*(N = 28)*	*(N = 25)*			
**Somatoform**	72.9 (63.4–82.3)	67.8 (50.4–85.1)	92.0 (81.4–100.0)			
**Major depression**	27.0 (17.6–36.4)	25.0 (8.9–41.0)	8.0 (2.6–18.6)			
**Other depression**	45.9 (35.3–56.4)	28.5 (11.7–45.2)	40.0 (20.7–59.2)			
**Anxiety**	31.7 (21.8–41.6)	3.6 (3.3–10.5)	4.0 (3.6–11.7)			
**PTSD**	8.2 (2.4–14.0)	3.6 (3.3–10.5)	4.0 (3.6–11.7)			

*Statistically significant differences in prevalence are indicated in bold font.

**Prevalence difference were not calculated due to small sample sizes.

Of 365 participants without CMD at baseline, 267 (73.0%) were successfully followed and incident CMD was present in 8 (2.2%). Of the 85 participants with CMD at baseline, 71 (83.5%) were followed, 51 (71.8%) of whom no longer had CMD. One year CMD incidence and maintenance were therefore 2.2% (95% CI 0.7–3.7) and 28.2% (18.6–37.7), but sample sizes were judged to be insufficient for analysis of factors associated with these outcomes.

Unadjusted RRR for any CMD between the group remaining in displacement and the returnee group was 1.2 (95% CI 0.7–2.1), and remained unchanged when adjusted for socio-demographic factors (1.2; 95% CI 0.6–2.2). Unadjusted associations between socio-demographic variables and CMD prevalence in the two follow-up samples are summarised in Table 3, with findings from the baseline sample reproduced for comparison. Older age was significantly associated with CMD in all samples, while a female excess was only significant at baseline and in the return migrant sample Widowed/divorced civil status on the other hand was only significantly associated with CMD in those remaining in displacement at follow-up. Food insecurity was most strongly associated with CMD in the return migrant group and there was no significant association with debt in any of the three samples. Social isolation was significantly associated with CMD in all three groups, with the strongest association in the group remaining in displacement. Land ownership prior to displacement and source of resettlement land were not significantly associated with CMD in those remaining in displacement (OR 1.7 95% CI 0.7–3.9) or return migrants (OR 1.9 95% CI 0.8–4.5) (data not shown).

**Table 3:** **Univariate unadjusted associations between CMD and socio-demographic characteristics in the three analysed groups**

Characteristic	Association with CMD (odds ratio, 95% CI)		
	1. Baseline sample (2011) N = 85/450	2. Previous participants still displaced in 2012 N =28/324	3. Return migrants recruited in 2012 N =25/242
**Age**			
*18–37 (born in/child at displacement)*	Reference	Reference	Reference
*38–65 (adult at displacement)*	**2.1 (1.6–2.7)**	**2.7 (1.2–6.2)**	**2.5 (1.0–6.3)**
**Gender**			
*Male*	Reference	Reference	Reference
*Female*	**1.6 (1.0–2.7)**	1.3 (0.6–3.2)	**4.4 (1.3–15.3)**
**Marital status**			
*Married*	Reference	Reference	Reference
*Widowed/Divorced*	**4.9 (2.4–9.9)**	**5.0 (1.9–12.9)**	1.6 (0.5–5.1)
*Never married*	**0.4 (0.1–0.9)**	1.2 (0.3–5.5)	0.3 (0.04–2.1)
**Education**			
*Primary/secondary (grade 5-upto OL)*	**2.3 (1.0–5.6)**	2.3 (0.5–9.9)	5.1 (0.7–38.8)
*Post-secondary (AL & above)*	Reference	Reference	Reference
**Employment**			
*Employed*	Reference	Reference	Reference
*Unemployed*	**2.8 (1.6–4.9)**	1.6 (0.7–3.8)	2.0 (0.8–5.2)
**Financial debt**			
*No debts*	Reference	Reference	Reference
*Indebted*	1.0 (0.6–1.6)	0.6 (0.2–1.6)	0.2 (0.03–1.8)
**Food security (last year)**			
*Sufficient food*	Reference	Reference	Reference
*Lack of sufficient food*	**1.7 (1.0–2.8)**	1.6 (0.7–3.8)	**6.5 (1.9–21.8)**
**Social network**			
*Adequate network*	Reference	Reference	Reference
*Social isolation*	**2.1 (1.2–3.7)**	**3.5 (1.4–8.4)**	**2.7 (1.0–7.1)**

Bold values are significant at p < 0.001.

Adjusted multivariable logistic regression analysis results are summarised in Table 4. Social isolation was significantly associated with CMD for both models in the group remaining in displacement, while female gender had strongest associations for both models in the return migrant group (albeit with limited precision).

**Table 4:** **Multivariate regression models of associations between demographic/economic/social network factors and CMD in the three analysed groups**

Characteristic						
	1. Baseline sample (2011)		2. Previous participants still displaced in 2012		3. Return migrants recruited in 2012	
	Model 1 ^ a ^ - Adj.	Model 2 ^ b ^ - Adj.	Model 1 ^ a ^ - Adj.	Model 2 ^ b ^ - Adj.	Model 1 ^ a ^ - Adj.	Model 2 ^ b ^ - Adj.
	OR (95% CI)	OR (95% CI)	OR (95% CI)	OR (95% CI)	OR (95% CI)	OR (95% CI)
**Age**						
*18–37 (born in/child at displacement)*	Reference					
*38–65 (adult at displacement)*	**1.9 (1.4–2.5)**	**1.8 (1.3–2.4)**	2.1 (0.8–5.3)	2.1 (0.8–5.4)	2.0 (0.7–5.8)	2.0 (0.7–5.8)
**Gender**						
*Male*	Reference					
*Female*	1.5 (0.8–2.6)	0.5 (0.2–1.2)	1.0 (0.4–2.6)	0.6 (0.2–2.4)	**5.1 (1.4–18.9)**	**5.2 (1.0–26.5)**
**Marital status**						
*Married*	Reference					
*Widowed/Divorced*	**2.5 (1.2–5.4)**	**2.8 (1.2–6.1)**	2.9 (0.9–8.8)	2.8 (0.9–8.6)	0.5 (0.1–2.1)	0.5 (0.1–2.1)
*Never married*	0.6 (0.2–1.8)	0.6 (0.2–1.7)	1.6 (0.3–8.1)	1.6 (0.3–8.0)	0.6 (0.1–6.0)	0.6 (0.1–6.1)
**Education**						
*Primary/secondary (grade 5-upto OL)*	1.2 (0.5–3.2)	1.1 (0.4–2.9)	1.6 (0.4–2.6)	1.6 (0.3–7.6)	3.1 (0.3–28.8)	3.1 (0.3–29.1)
*Post-secondary (AL & above)*	Reference					
**Financial debt**						
*No debts*	Reference					
*Indebted*	0.9 (0.5–1.6)	0.9 (0.5–1.6)	0.4 (0.1–1.4)	0.4 (0.1–1.5)	0.2 (0.03–1.7)	0.2 (0.03–1.7)
**Food security (last year)**						
*Sufficient food*	Reference					
*Lack of sufficient food*	**1.9 (1.1–3.2)**	**1.9 (1.1–3.4)**	1.9 (0.6–5.7)	2.0 (0.7–6.0)	3.5 (0.8–14.4)	3.4 (0.8–14.9)
**Social network**						
*Adequate network*	Reference					
*Social isolation*	**2.0 (1.1–3.7)**	**1.9 (1.0–3.5)**	**2.9 (1.2–7.2)**	**3.0 (1.2–7.4)**	1.4 (0.5–4.2)	1.4 (0.5–4.2)
**Employment**						
*Employed*	Reference					
*Unemployed*	**-**	**4.0 (1.7–9.3)**	-	1.8 (0.5–6.4)	**-**	1.0 (0.3–3.7)

^a^Adjusted for all variables in table except for employment.

^b^Adjusted for all variables in table including employment.

Bold values are significant at p<0.001.

## Discussion

As the first study to explore changes over time in mental health of IDP after prolonged internal displacement in Sri Lanka, to our knowledge this is also one of the first of its kind in the more global context of conflict-driven internal migration. Existing evidence on returnees has focused on returned refugees rather than returned IDPs [26,27], with the exception of a study in Georgia that only used a cross-sectional design [28]. Our study followed a sample of IDPs in North-Western Sri Lanka, displaced in 1990 and in the process of choosing between remaining in the displacement region or returning to areas of origin after the cessation of conflict in 2009. The study incorporated a longitudinal investigation of changes in mental disorder prevalence among a cohort of IDP, affected by two decades of internal displacement and considering post-conflict resettlement, supplementing this with additional data from people from the same communities who had relocated.

The findings show that during the relatively short period between the baseline and follow-up studies, mental disorder prevalence had substantially decreased. Overall CMD prevalence had more than halved from 18.8% to 8.6% in the follow-up group remaining in displacement. This is not explained by selective attrition, since those with CMD at baseline were more rather than less likely to be followed, and is not explained by measurements which were identical on both occasions. The prevalence in the supplementary sample of return migrants was comparably low which supports a genuine reduction in prevalence since both groups were systematically sampled with 100% participation. In addition, there were demographic and socioeconomic similarities between the baseline and supplementary returnee samples supported the latter’s comparability. A significant decrease in PTSD prevalence (23.2% in 2001 against 14.5% in 2007) was also found in a post-war, 6-year follow-up in Kosovo Albanians [11], although clearly our findings indicate higher reductions over a much shorter period. A cross-sectional study in Georgia comparing mental health outcomes of IDPs and returnees also found significantly lower levels of PTSD, depression, anxiety and co-morbidity among returnees than IDPs [28]. Among the constituent disorders, somatoform disorder was most associated with the reduction in prevalence. Prevalence of PTSD at baseline (2.8%) was lower than that generally reported for other global IDP populations [16] and even lower at the follow-up stage. Time elapsed since trauma, longer duration of displacement, relative peace in the areas surrounding the displaced settlements, and being born in displacement, might have contributed [16].

The decreased overall prevalence of CMD in this cohort is unlikely to be directly related to the cessation of conflict, since this occurred 2 years before the baseline study and the Puttalam region was largely spared the conflict-related violence experienced elsewhere. However, it might be attributed to several other factors. The simple opportunity to return to an area of origin may have precipitated an improvement in mental health, despite the fact that relatively few (3.1%) of the followed sample had done so. This may reflect a general feeling of freedom, or more specific prospects of returning to a preferred way of life and/or work, perceived economic or other advantages, and reduced discrimination. However, it is also possible that factors such as levels of acculturation among generations born in displacement, and socio-economic improvements (as evidenced by less debt, unemployment and food insecurity in the returning group in this study), may have influenced the resulting prevalence changes. In addition, access to mental health services may have increased after the cessation of conflict, as resources are re-allocated during the peacetime rebuilding efforts. More infrastructure development has taken place during the post-conflict areas, while more medical resources, including those for mental health, have been allocated for IDP populations. Although there have been no specific interventions targeting the IDP populations, limited psychosocial support services have become available. These factors may also have played a role in improvement of mental health in these populations, although it is clearly not possible to infer a direct cause with certainty from observational data.

Unadjusted analyses showed similar associations between CMD and factors such as older age group (displaced as adults), female gender, widowed/divorced status, financial debt and lack of food security as found in the baseline phase, although not uniform, across both follow-up groups [16]. In this respect, the return migrant group characteristics had more in common with the baseline sample than those remaining in displacement. In another study conducted among post-conflict displaced populations in Sri Lanka, recently resettled groups were more likely to report symptoms of trauma [29]. Although studies conducted among refugees support the fact that staying within stable, favourable environments coupled with higher acculturation predicts better mental health outcomes, there is insufficient evidence among IDPs due to the dearth of empirical evidence [2]. Factors such as source of resettlement land (owned or government-provided) in areas of return have may have an effect on the mental health outcomes of the IDP population, as previous evidence shows that land related issues play an important role in the return migration process [6,7]. However, no such associations were found in this particular Sri Lankan sample.

Female gender was strongly associated with CMD in the return migrant group after adjustment. In the context of the studied population, this may be linked to the experience of increased difficulties during the return process, especially for female-headed households or where there are links to employment. As the main livelihoods of the IDP community in question are fishing or farming, female participants might have been experiencing difficulties finding employment in areas of origin, especially as post-conflict areas lack economic resources or ready-made job opportunities. Previous studies have found that female gender is associated with higher levels of stress related to low economic opportunities in conflict-affected situations [11,30]. Husain et al. [2011] in a recent study looking at mental health and displacement in Jaffna district of Northern Sri Lanka also found an association between female gender and poor mental health, which may highlight cultural and conflict-related contextual similarities [29]. The study of IDPs and returnees in Georgia noted above also found that female gender was associated with CMD among returnees [28].

Social isolation or lack of social networks were significantly associated with CMD across all three groups in unadjusted analyses and significantly associated with CMD at baseline and in those remaining in displacement after adjustment. Availability of adequate social networks is seen as a protective factor against the development of mental illnesses, especially for those populations who experience traumatic migration [31]. However, empirical data on social isolation and mental disorder prevalence among internally displaced populations are lacking [32] with little or no investigation of the impact of social networks on mental health, among returning IDPs. Our findings indicate that social isolation might be an important factor associated with mental disorders among forced internal migrants, especially those affected by protracted displacement. Interestingly, social isolation was not found to be associated with CMD in the return migrant group. This may be due to other socio-demographic factors influencing or curtailing social networks while in displacement or after return migration [32], or might be because there are lower expectations of social networks following recent return migration. Further empirical research is needed.

As mentioned before, most studies conducted on returning forced migrant samples are focused on exploring development-related issues and have provided little data on health-related outcomes [8]. Although understanding issues related to security, land provision, livelihoods and other infrastructure in ensuring successful return for displaced IDPs is important, understanding health-related issues is also important in order to foster a healthy migrant population and the subsequent establishment of better integrated communities in post-conflict areas [6]. Factors identified in this study as associated with CMD, such as female gender and social isolation, are likely to be strongly related to the development-related processes mentioned above. In addition, generations born in displacement, without a clear and distinct bond to the areas of origin of their IDP parents require special attention to understand specific mental health issues that may arise through having to move to an unfamiliar place, mainly in order to keep family cohesion or simply due to lack of choice.

Armed conflict is recognised as a major public health challenge in the current global context particularly in relation to mental disorders and especially for resource-poor developing countries [33]. In post-conflict settings, having an effective mental health system in place is crucial for reducing the disease burden associated with conflict related trauma experienced by the majority of IDP [34,35]. Sri Lanka has been cited as a case study for strengthening the health system and addressing health-related risk factors for returning forced internal migrants although most activities in this regard have focused on physical health and addressing immediate logistics issues, rather than focus on longer-term mental health [6]. The northern districts of Sri Lanka are undergoing rapid reconstruction of health systems and services during the post-conflict era and both mental health problems in these populations and lack of adequate mental health care, are key priorities [36]. It is hoped that the findings on mental disorder prevalence and the longitudinal data presented in this paper will contribute substantially to the evidence base on return migration both in Sri Lanka and internationally.

### Strengths and limitations

This study has several strengths. It reports longitudinal, follow-up data on a group of IDPs experiencing prolonged displacement and subsequent return migration, which are virtually absent in the existing literature. Participation at baseline and in the supplementary sample of return migrants was 100%, attained because of the careful building up of a close rapport with the IDP community, demonstrating the feasibility of such research among difficult to reach, vulnerable groups in resource-poor post-conflict settings. Follow-up was also relatively high and, as stated, was higher rather than lower in people with CMD at baseline, thus not explaining a decline in prevalence.

Among its limitations, the high fluidity and internal migration in post-conflict Sri Lanka is likely to have affected the follow-up rate. Although at the time of this study, relatively few IDPs (including from the baseline cohort) had fully returned to areas of origin, recent information indicate that return rates have rapidly increased after the conclusion of data collection [37] and the situation remains fluid. The relatively small number of returnees followed-up from the baseline cohort may have limited us from obtaining a clearer picture of predictors of return migration. Other limitations in the study include the female preponderance in the baseline sample and limited sample size for some analyses. Both the baseline and follow-up studies were conducted more than 20 years since the displacement event, and the selective representation of the displacement time frame may have had an impact on the findings. The instruments chosen have been used previously in the same IDP population in Sri Lanka and other cross-cultural settings, although issues arising from varied measurements are recognised [5]. A novel method that includes nominal group techniques was used to adapt these measures to Sri Lankan context, in order to avoid cross-cultural usage biases [21]. This study used standard definitions of CMD and related constructs, and we acknowledge that there is a considerable debate about the trans-cultural relevance of such definitions and also about the differentiation between CMD and what might be termed situational distress. The cross-cultural relevance of mental health constructs derived from Western settings should always receive consideration, although it is important to bear in mind that Sri Lankan public health and clinical practice have followed European models since the 19th century. Female preponderance in the sample reflects the source population characteristics, and other studies in post-conflict areas of Sri Lanka have shown high proportions of female participants [29], in common with the baseline COMRAID study [16]. The follow-up period of one year may not have been adequate to measure the natural progression or remission of CMD, thus limiting the inferences about actual reasons for recovery or continuity of disorder status. In addition, the study design does not enable us to show causation, and reverse causality should be recognised for some associations such as social isolation and CMD. Another limitation may be the exclusion of trauma exposure in the regression models. This was considered but was found to be collinear with age (i.e. generational differences). The approach adopted in this study was epidemiological and quantitative. Further insights into displacement and return migration experiences would clearly be gained through supplementary qualitative methods.

## Conclusions

This is the first study exploring the changes in mental health of an IDP population affected by conflict-driven prolonged forced displacement, in the process of return migration in Sri Lanka. It shows a clear decrease in CMD prevalence among this population and highlights socio-demographic factors associated with prevalence. The socio-economic associations, similar to those found in other global IDP studies, highlight commonalities in factors associated with mental disorders across diverse cultures and geographical settings. These findings underline the need to address socio-economic stressors as well as mental health service requirements in a co-ordinated manner among the IDP populations. A systematic review by Roberts & Browne [3] identified a clear need to understand the changes in mental health of conflict-affected in low and middle income countries, particularly among post-conflict returnees [4]. Findings presented here add important evidence to this existing gap in knowledge, and stand to aid policy development and service provision during post-conflict resettlement both in Sri Lanka and other global IDP situations, as well as strengthening the need for a broader public mental health approach to internal displacement. For an example, findings from the baseline study have already led to the development of an intervention that aims to integrate mental health into primary care in both Puttalam and Mannar districts, by training primary care practitioners catering to IDP populations to identify, treat and refer CMD patients with increased effectiveness [38]. This intervention is oriented around the World Health Organization mental health Gap Action Programme (WHO mhGAP), and promotes a process of broadening research and treatment development from a current narrow focus on trauma and PTSD, adapting a public health oriented intervention development strategy that focuses on a wider spectrum of mental disorders.
